# H19- and hsa-miR-338-3p-mediated NRP1 expression is an independent predictor of poor prognosis in glioblastoma

**DOI:** 10.1371/journal.pone.0260103

**Published:** 2021-11-29

**Authors:** Yong Liu, Yuelin Liu, Yong Gao, Lei Wang, Hengliang Shi, Chengmin Xuan

**Affiliations:** 1 Department of Neurosurgery, Brain Hospital, Affiliated Hospital of Xuzhou Medical University, Xuzhou, Jiangsu, P.R. China; 2 Department of Clinical Medicine, Xuzhou Medical University, Xuzhou, Jiangsu, P.R. China; 3 Department of Orthopaedics, Xuzhou Children’s Hospital of Xuzhou Medical University, Xuzhou, Jiangsu, P.R. China; 4 Central Laboratory, Affiliated Hospital of Xuzhou Medical University, Xuzhou, Jiangsu, P.R. China; 5 Medical Section, Xuzhou Children’s Hospital of Xuzhou Medical University, Xuzhou, Jiangsu, P.R. China; University of Ulsan College of Medicine, REPUBLIC OF KOREA

## Abstract

Glioblastoma multiforme (GBM) is the most common and also the most invasive brain cancer. GBM progression is rapid and its prognosis is poor. Therefore, finding molecular targets in GBM is a critical goal that could also play important roles in clinical diagnostics and treatments to improve patient prognosis. We jointly analyzed the GSE103227, GSE103229, and TCGA databases for differentially expressed RNA species, obtaining 52 long non-coding RNAs (lncRNAs), 31 microRNAs (miRNAs), and 186 mRNAs, which were used to build a competing endogenous RNA network. Kaplan–Meier and receiver operating characteristic (ROC) analyses revealed five survival-related lncRNAs: H19, LINC01574, LINC01614, RNF144A-AS1, and OSMR-AS1. With multiple optimization mRNAs, we found the H19-hsa-miR-338-3P-NRP1 regulatory pathway. Additionally, we noted high NRP1 expression in GBM patients, and Kaplan–Meier and ROC analyses showed that NRP1 expression was associated with GBM prognosis. Cox analysis indicated that NRP1 is an independent prognostic factor in GBM patients. In conclusion, H19 and hsa-miR-338-3P regulate NRP1 expression, and this pathway plays an important role in GBM.

## Introduction

Glioblastoma multiforme (GBM) is the most malignant pathological type of glioma. GBMs grow rapidly, are highly malignant, and generally progress rapidly, leading to an average survival period of approximately 12 months [1, 2]. Although comprehensive treatments including surgery, radiotherapy, and chemotherapy have recently improved, the survival time of GBM patients remains unsatisfactory [3, 4]. Currently, tumor genomics provides a basis for discovering pathogenic alterations in malignant cells that can become therapeutic targets, offering hope for GBM patients [5]. Our early bioinformatics analysis also found molecules related to the prognosis of glioma patients and ubiquitinated molecules related to the prognosis of glioma [3, 5].

Long non-coding RNAs (lncRNAs), which are an RNA species of greater than 200 nt in length that lack protein coding functions, account for the majority of transcribed sequences in the human genome [6–8]. LncRNAs act as competitive endogenous RNAs (ceRNAs), binding to miRNAs and “sponging” them from their targets, thereby regulating the protein levels of coding genes [3, 9–11]. Recent studies have shown that the dysregulation of lncRNAs, microRNAs (miRNAs), and their downstream target genes plays an important role in the occurrence and development of tumors [12–14]. However, the specific mechanisms of lncRNAs as ceRNAs in individual tumor types remain to be discovered.

NRP1 is a non-tyrosine kinase transmembrane protein composed of 923 amino acids that is widely expressed in diverse tissue types [15]. NRP1 is also expressed in a variety of tumors including leukemia, prostate cancer, breast cancer, pancreatic cancer, and glioma [16, 17]. Studies have shown that NRP1 regulates vascular endothelial factor (VEGF) to promote tumor angiogenesis [18, 19]. NRP1 interacts with integrin beta-1 in pancreatic ductal adenocarcinoma to promote tumor growth and invasion [20]. Although we have conducted some studies on the role of NRP1 in tumors, we know very little about its role in GBM [21, 22]. Therefore, we analyzed the relationship between NRP1 and the prognosis of GBM using genomics.

We obtained differentially expressed lncRNAs and mRNAs by analyzing GBM databases, predicted the miRNAs that could interact with them, and then constructed a lncRNA-miRNA-mRNA regulatory network. Kaplan–Meyer and receiver operating curve (ROC) analyses revealed five survival-related lncRNAs: H19, LINC01574, LINC01614, RNF144A-AS1, and OSMR-AS1. Additionally, we found that NRP1, HOXC6, and SHCBP1 mRNAs were differentially expressed in GBM and were related to patient prognosis. NRP1 is highly expressed in GBM, and it was confirmed in the Chinese Glioma Genome Atlas (CGGA) and GSE16011 databases that NRP1 expression is related to patient prognosis. Cox analysis confirmed that NRP1 was an independent prognostic factor for GBM patients. Ultimately, we propose that the H19-hsa-miR-338-3P-NRP1 pathway could be targeted for GBM treatment and/or used for clinical diagnostics.

## Materials and methods

### Patient samples

The lncRNA and mRNA data of GBM patients were obtained from the Gene Expression Omnibus (GEO), CGGA, and The Cancer Genome Atlas (TCGA) databases. GSE103227 (https://www.ncbi.nlm.nih.gov/geo/query/acc.cgi?acc=GSE103227) and GSE103229 (https://www.ncbi.nlm.nih.gov/geo/query/acc.cgi?acc=GSE103229) each contain five non-tumor brain specimens and five GBM specimens. The TCGA (https://www.cancer.gov/about-nci/organization/ccg/research/structural-genomics/tcga) database contains five non-tumor brain specimens and 168 GBM specimens. GSE16011 (https://www.ncbi.nlm.nih.gov/geo/query/acc.cgi?acc=GSE16011) contains eight non-tumor brain specimens and 148 GBM specimens. CGGA (http://cgga.org.cn/) contains 108 GBM patients. We download glioblastoma-related data according to the steps provided on these websites. Xuzhou Children’s Hospital Medical Ethics Committee approved this study protocol.

### Obtaining differentially expressed lncRNAs and mRNAs and constructing the ceRNA network

We obtained differentially expressed lncRNAs and mRNAs using the LIMMA package of R software (www.r-project.org/). We defined differentially expressed lncRNAs and mRNAs as those that satisfy both |logFC| ≥1 and p-value <0.05. Then, Venn diagrams were used to display the intersection of the identified lncRNAs and mRNAs from each dataset. Then miRcode software (http://www.mircode.org/) was used to predict miRNAs that could interact with the identified lncRNAs. MiRNAs that concurrently satisfied miRDB (http://mirdb.org/), miRTarBase (https://bio.tools/mirtarbase) and TargetScan (http://www.targetscan.org/vert_72/) were defined as miRNA target genes. Finally, Cytoscape (https://cytoscape.org/) was used to generate the ceRNA network of lncRNA-miRNA-mRNA interactions.

### Expression of the lncRNAs and mRNAs and ROC, Kaplan–Meyer, and Cox analyses

The beeswarm package of R software was used to analyze the differential expression of NRP1. The survival and survivalROC packages of R software were used to perform Kaplan–Meyer and ROC analyses, respectively, of the lncRNAs and mRNAs. The survival and survminer packages of R software were used to perform Cox analysis.

### Statistical analysis

LncRNAs and mRNAs with |logFC| ≥1 and p-value <0.05 were considered statistically significant. ROC values >0.65 were defined as statistically significant. Univariate and multivariate Cox analyses were performed using R software, with *P*<0.05 considered statistically significant.

## Results

### Constructing the ceRNA network in GBM patients

**Fig 1:**
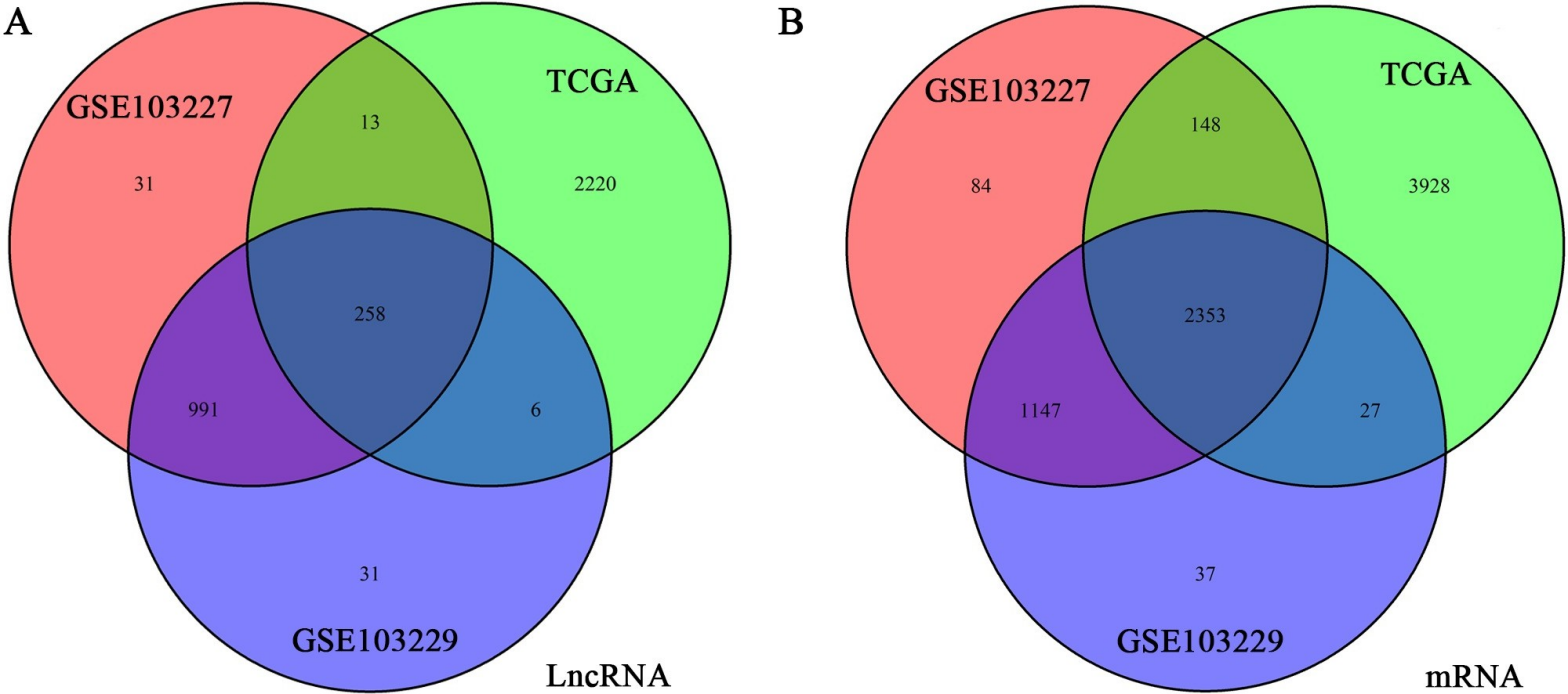
Acquisition of differentially expressed lncRNAs and miRNAs. (a, b) We obtained differentially expressed lncRNAs (a) and miRNAs (b) from the GSE103227, GSE103229, and TCGA databases. The Venn diagrams display the number of differentially expressed transcripts and the number of intersections.

**Fig 2:**
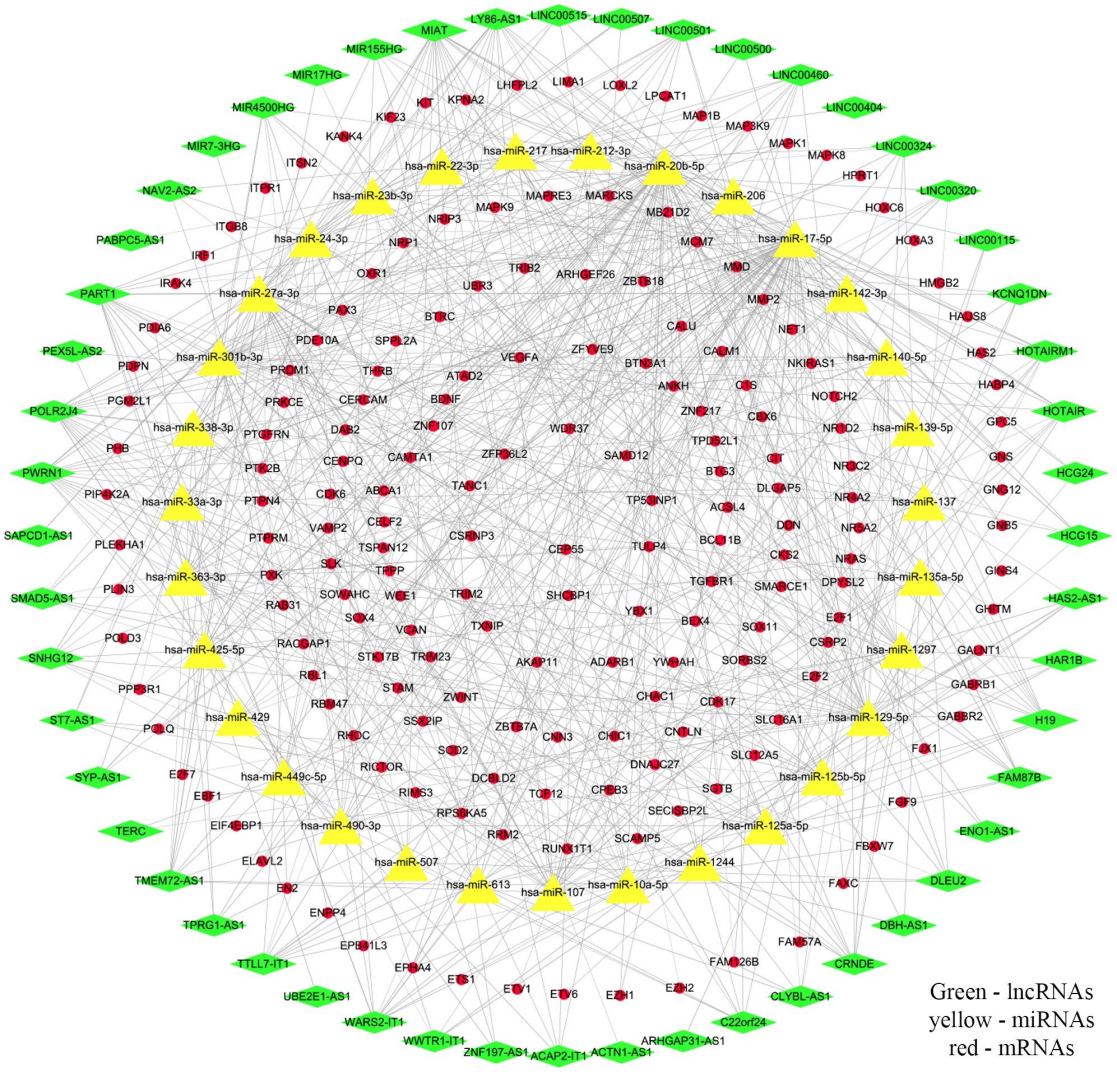
Constructing the ceRNA network from lncRNA and miRNA data. The interaction relationships between lncRNAs, miRNAs, and mRNAs were used to construct the ceRNA network. Green represents the 52 lncRNAs, yellow represents the 31 miRNAs, and red represents the 186 mRNAs.

To obtain reliable lncRNAs, miRNAs, and mRNAs associated with GBM patient prognosis, we jointly analyzed the GSE103227, GSE103229, and TCGA databases. These three datasets revealed 1293, 1286, and 2497 differentially expressed lncRNAs, respectively, with 258 at the intersection of the groups (Fig 1A). From these databases, we also obtained 3732, 3564, and 6456 differentially expressed mRNAs, with 2353 common to all datasets (Fig 1B). MiRNAs that could potentially interact with these lncRNAs were obtained using miRcode. And then we retain the interacting lncRNAs, miRNAs and mRNAs. These data were used to demonstrate interactions, which were compiled into the ceRNA network. In total, the network comprised 52 lncRNAs, 31 miRNAs, and 186 mRNAs (Fig 2, S1 Table).

### Obtaining mRNAs associated with GBM prognosis

**Fig 3:**
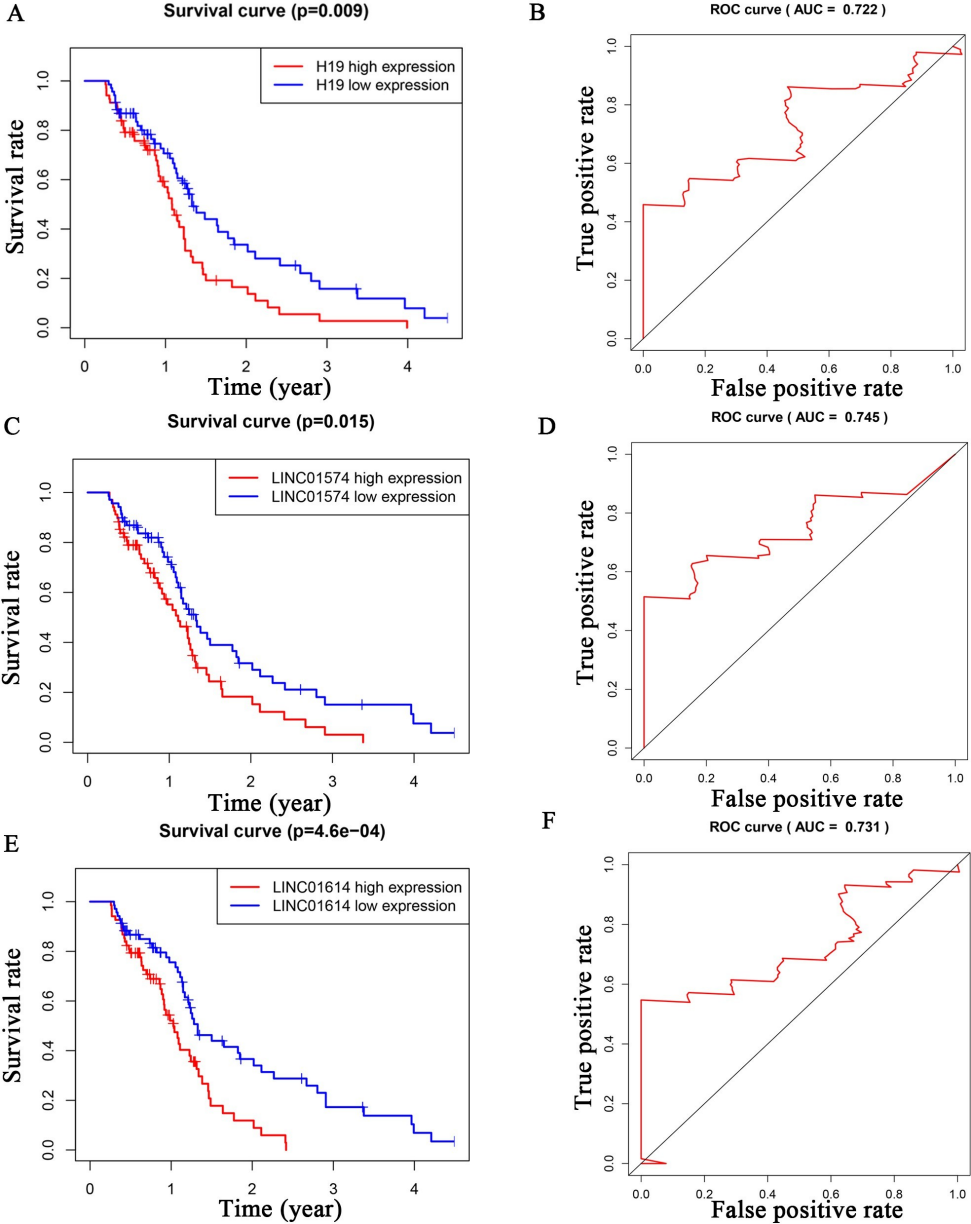
Kaplan–Meyer and ROC analyses of survival-related lncRNAs (H19, LINC01574, LINC01614). We examined the differentially expressed lncRNAs by Kaplan–Meyer and ROC analyses. The lncRNAs with p-values <0.05 and ROC values >0.7 were H19 (Fig 3A and 3B), LINC01574 (Fig 3C and 3D), LINC01614 (Fig 3E and 3F).

**Fig 4:**
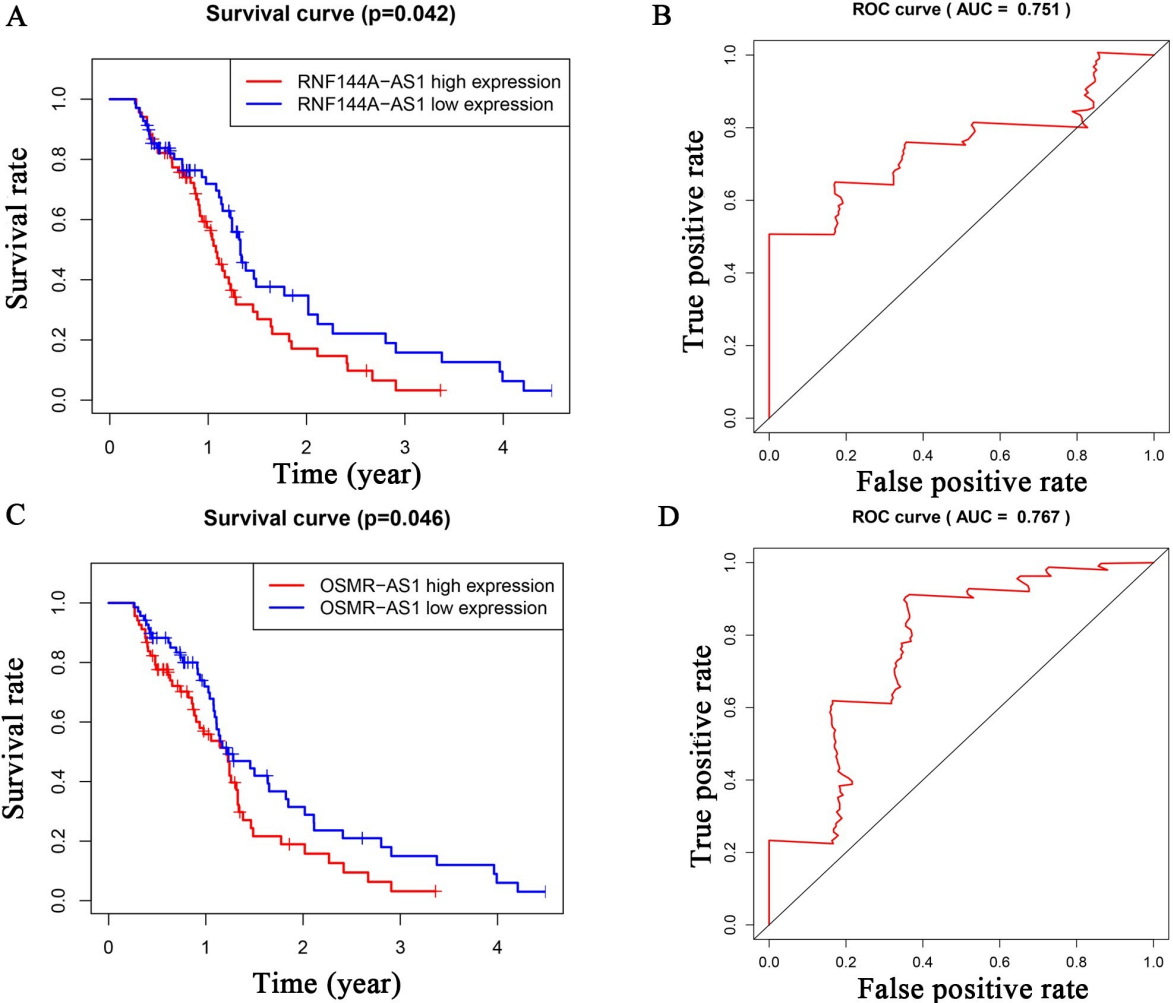
Kaplan–Meyer and ROC analyses of survival-related lncRNAs (RNF144A-AS1, OSMR-AS1). Kaplan-Meyer and ROC analyzed the relationship between lncRNAs and patient survival. We show two other statistically significant lncRNAs: RNF144A-AS1 (Fig 4A and 4B) and OSMR-AS1 (Fig 4C and 4D).

**Fig 5:**
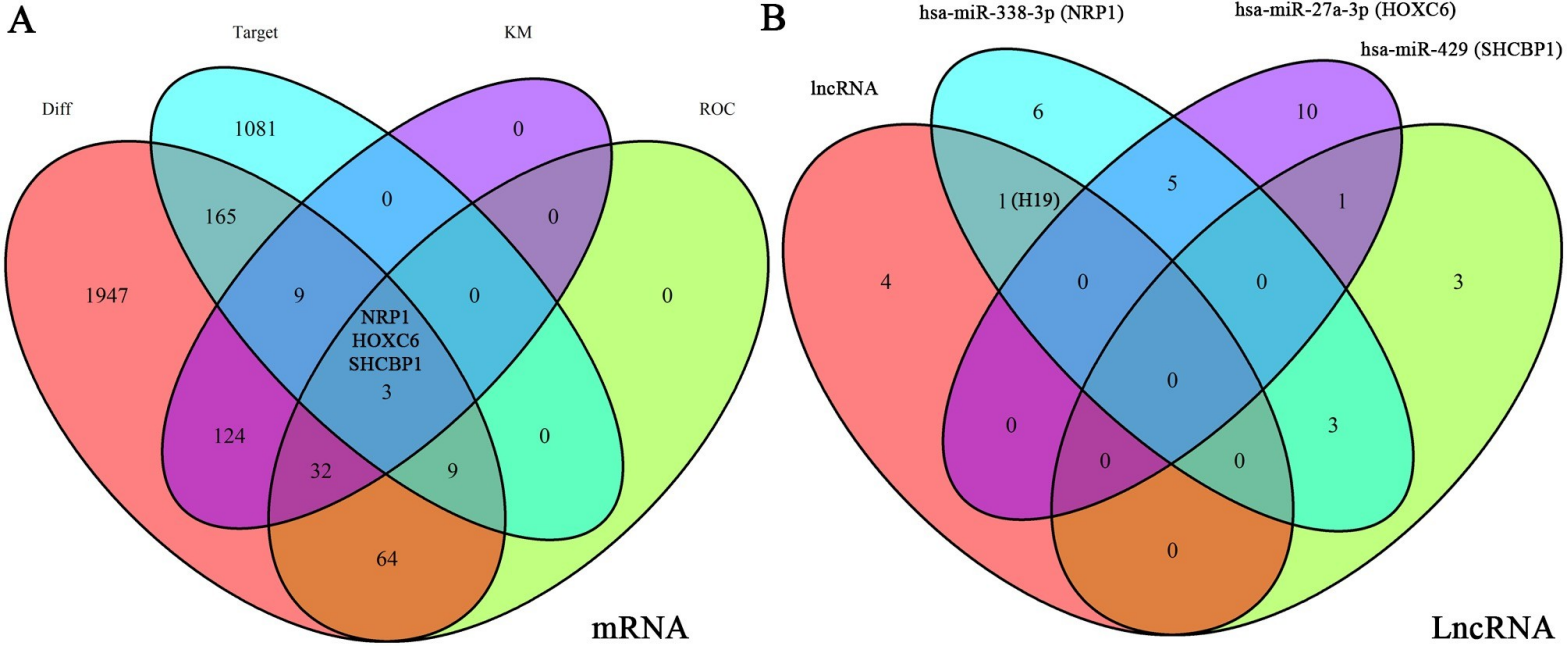
Screening for differentially expressed lncRNAs and miRNAs. (a) The intersection of the differentially expressed mRNAs, miRNAs predicted to bind these mRNAs, Kaplan–Meyer analysis, and ROC analysis was to find meaningful mRNAs, of which there were three: NRP1, HOXC6, SHCBP1. (b) We analyzed the intersection of the five survival-related lncRNAs with the corresponding lncRNAs of NRP1, HOXC6, and SHCBP1, which revealed the H19-hsa-miR-338-3p-NRP1 signaling axis. At the same time, we show miRNAs that correspond with the three mRNAs.

To investigate the lncRNAs closely associated with GBM prognosis, we plotted Kaplan–Meyer and ROC curves for the 52 lncRNAs. We found that H19, LINC01574, LINC01614, RNF144A-AS1, and OSMR-AS1 had p-values <0.05 and ROC values >0.7 (Figs 3 and 4). By obtaining the intersection of differential expression data from GSE103227, GSE103229 and TCGA (Diff), ROC data (mRNA with ROC values >0.7 among Diff), target data (mRNAs predicted by the miRNAs), and Kaplan–Meyer curves (mRNAs with p values <0.05 among Diff), we obtained more specific mRNA candidates: NRP1, HOXC6, and SHCBP1 (Fig 5A). The miRNAs predicted to interact with these three mRNAs included hsa-miR-338-3p (NRP1), hsa-miR-27a-3p (HOXC6), and hsa-miR-429 (SHCBP1) (Fig 5B). Among the above five lncRNAs, only H19 was predicted to interact with hsa-miR-338-3p (Fig 5B). Using this approach of combining multiple database analysis, we identified the H19-hsa-miR-338-3p-NRP1 signaling pathway in GBM patients.

### Cox analysis of using NRP1 to predict GBM patient prognosis

**Fig 6:**
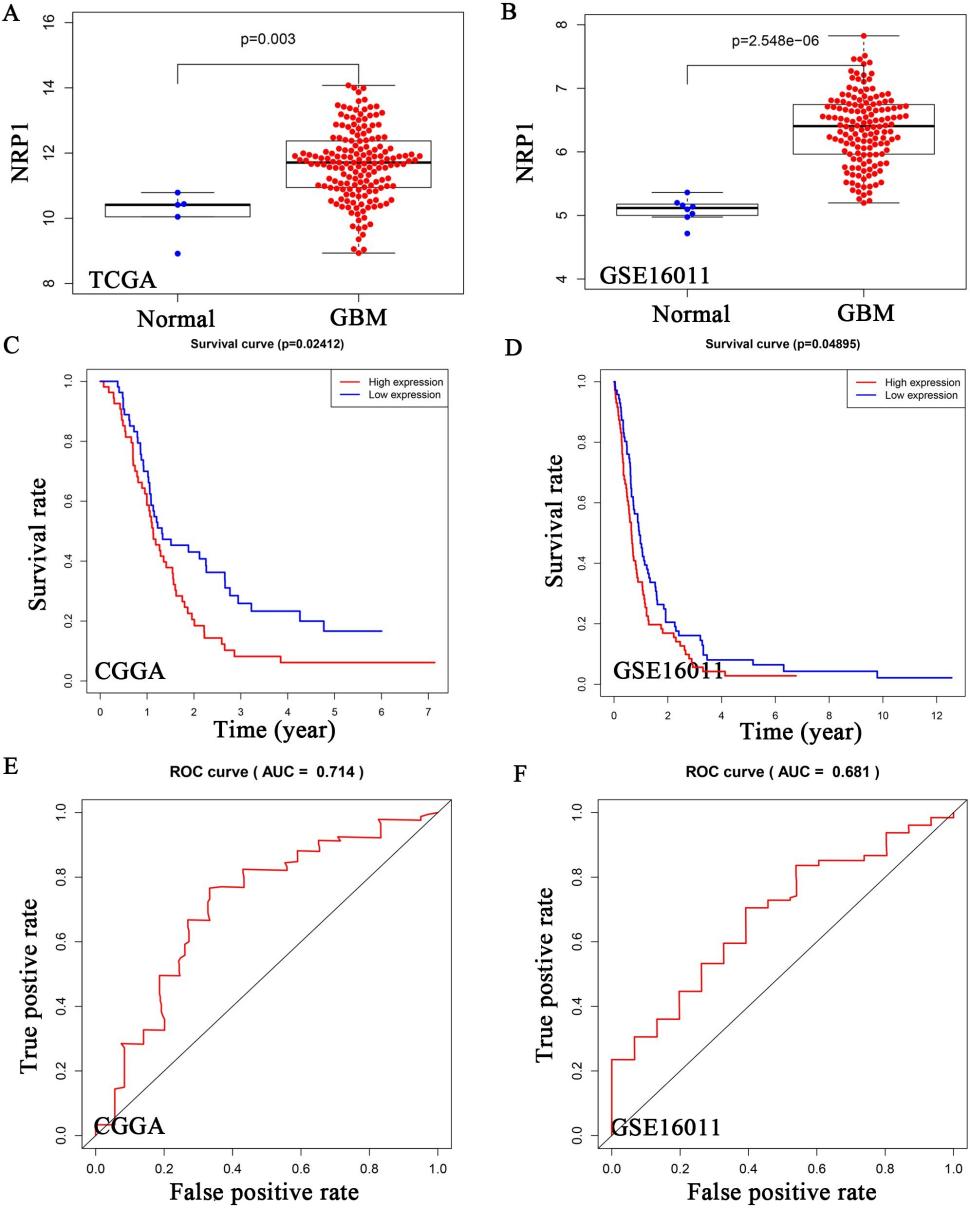
*NRP1* expression in gliomas and the relationship with prognosis. (a, b) TCGA and GSE16011 databases were used to show NRP1 expression in non-tumor brain tissues and GBM samples. (c-f) Kaplan–Meyer analysis (c, e) and ROC analysis (d, f) were performed in TCGA and GSE16011 databases.

**Fig 7:**
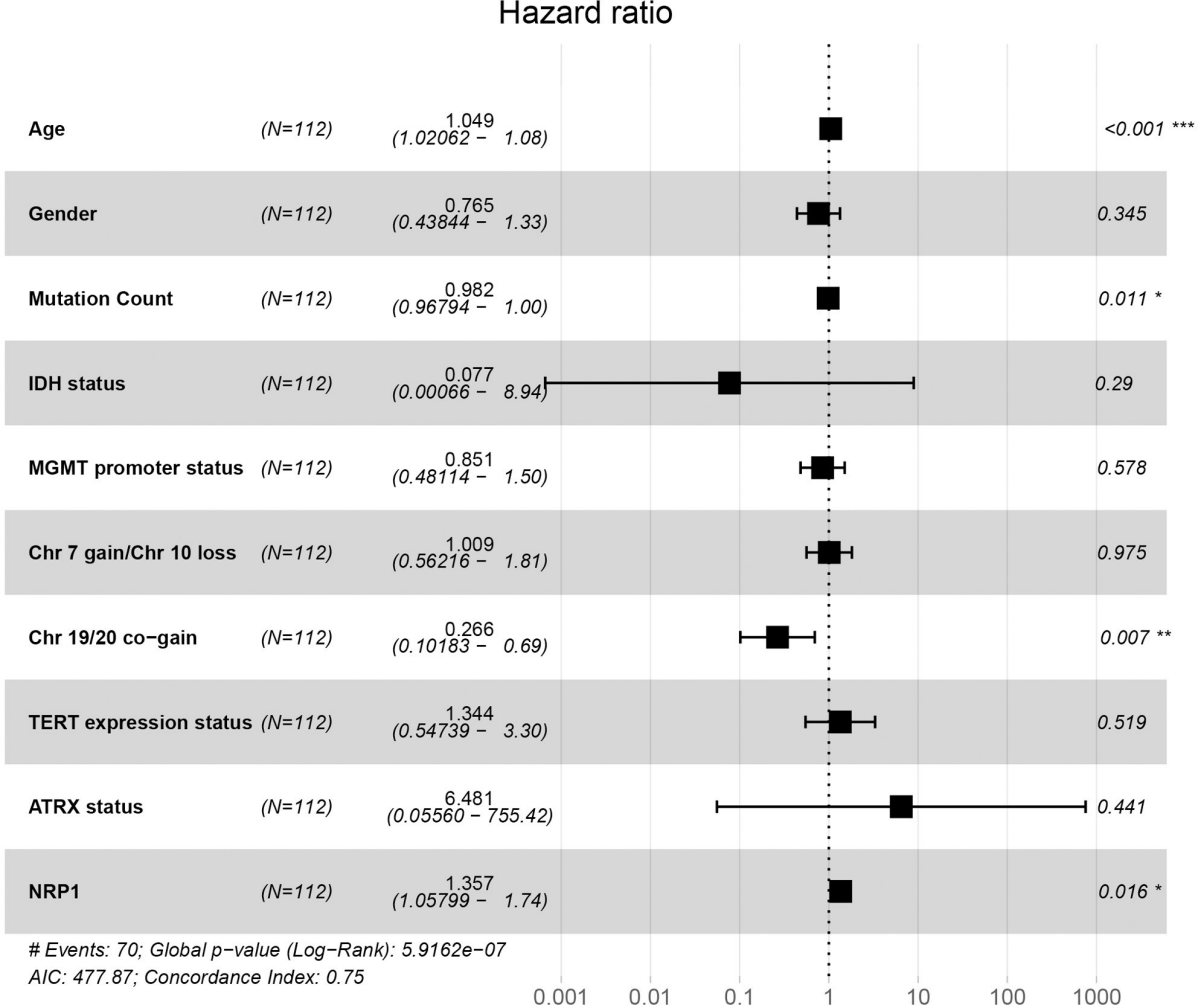
Multivariate Cox analysis of *NRP1*. NRP1 and multiple GBM features were used to analyze whether NRP1 was an independent prognostic factor for GBM.

In TCGA and GSE16011 datasets, we found that NRP1 was highly expressed in GBM (Fig 6A and 6B). Both Kaplan–Meyer and ROC analyses of NRP1 were statistically significant (Fig 6C–6F). Univariate Cox analysis found that age, IDH status, TERT status, ATRX status, and NRP1 were related to GBM patient prognosis, and the risk value increased by 1.052, 0.144, 0.582, 2.936, 0.180, and 1.294, respectively, for each additional unit (S2 Table). Multivariate Cox analysis found that age and NRP1 were independent prognostic factors for GBM patients, for which the patient risk value increased by 1.049 and 1.357 for each additional unit (Fig 7 and S2 Table). Using the newly constructed lncRNA-miRNA-mRNA regulatory network, we propose that NRP1 expression plays an important role in GBM and is regulated by H19 and has-miR-338-3p.

## Discussion

Through joint analysis of GSE103227, TCGA, and GSE103229 data, we obtained 258 differentially expressed lncRNAs and 2353 differentially expressed mRNAs. MiRcode was used to predict miRNAs that could interact with the obtained lncRNAs; then mRNAs that could interact with these miRNAs were predicted. We constructed a ceRNA regulatory network using the list of interacting lncRNAs, miRNAs, and mRNAs, for which there were 52, 31, and 186, respectively. Kaplan–Meyer and ROC analyses revealed five survival-related lncRNAs: H19, LINC01574, LINC01614, RNF144A-AS1, and OSMR-AS1. To improve the accuracy of our data, we obtained three mRNAs through limited conditions: NRP1, HOXC6, and SHCBP1. Using these three mRNAs, we found the miRNAs that could potentially act on them, and then obtained the lncRNAs that act on these miRNAs. The intersection of these analyses revealed that only H19 satisfied all conditions. Therefore, we propose that NRP1 is regulated by a ceRNA network consisting of H19 and hsa-miR-338-3p. In TCGA and GSE16011, NRP1 was found to be highly expressed in GBM, and in CGGA and GSE16011, it was confirmed by both Kaplan–Meyer and ROC analyses that NRP1 was significantly associated with GBM outcomes. Cox analysis asserted that NRP1 was an independent prognostic factor for GBM.

Previous studies have shown that H19 promotes cancer development through different mechanisms, as examples: in gastric cancer through Fas-related protein, in cholangiocarcinoma through IL-6 and CXCR4, in colorectal cancer through HMGA1, and in multiple myeloma through P50/P65 [23–26]. Hsa-miR-338-3p is a tumor suppressor gene in glioma and colorectal cancer but an oncogene in salivary adenoid cystic carcinoma [27–30]. NRP1 is not only closely related to the occurrence and development of tumors and tumor immunity, but also related to vascular development [31–36]. However, we still do not know the lncRNAs and miRNAs that interact with NRP1 or whether NRP1 is an independent prognostic factors for GBM patients. On the basis of our analyses of a large number of GBM specimens from multiple databases, we have revealed the interactions of H19 and hsa-miR-338-3p with NRP1, and have shown that NRP1 is an independent factor for the prognosis of GBM patients. In addition, considering our early discovery of glioma, we found that the mechanism of glioblastoma has its own uniqueness [3]. However, how NRP1 regulated by H19 and hsa-miR-338-3p regulates the occurrence and development of GBM still needs further research.

In conclusion, we found that NRP1 is closely related to the prognosis of GBM patients and is an independent prognostic factor. NRP1 is highly expressed in GBM. In GBM, H19 and hsa-miR-338-3p regulate NRP1, and this signaling pathway may serve as an important molecular target for the diagnosis and treatment of GBM patients, thereby improving the prognosis of GBM.

## Supporting information

S1 Table List of lncRNA, miRNA and mRNA in ceRNA. (XLSX)

S2 Table Univariate analysis and multivariate analysis of the correlation of the expression of NRP1 with OS among GBM patients. (PPTX)
