# Supplementary material for: H19- and hsa-miR-338-3p-mediated NRP1 expression is an independent predictor of poor prognosis in glioblastoma
Source: PLoS One. 2021 Nov 29;16(11):e0260103. doi: 10.1371/journal.pone.0260103 (PMC8629300; doi:10.1371/journal.pone.0260103)
Supplement: S2 Table — (PPTX) [file pone.0260103.s002.pptx]

## Slide 1
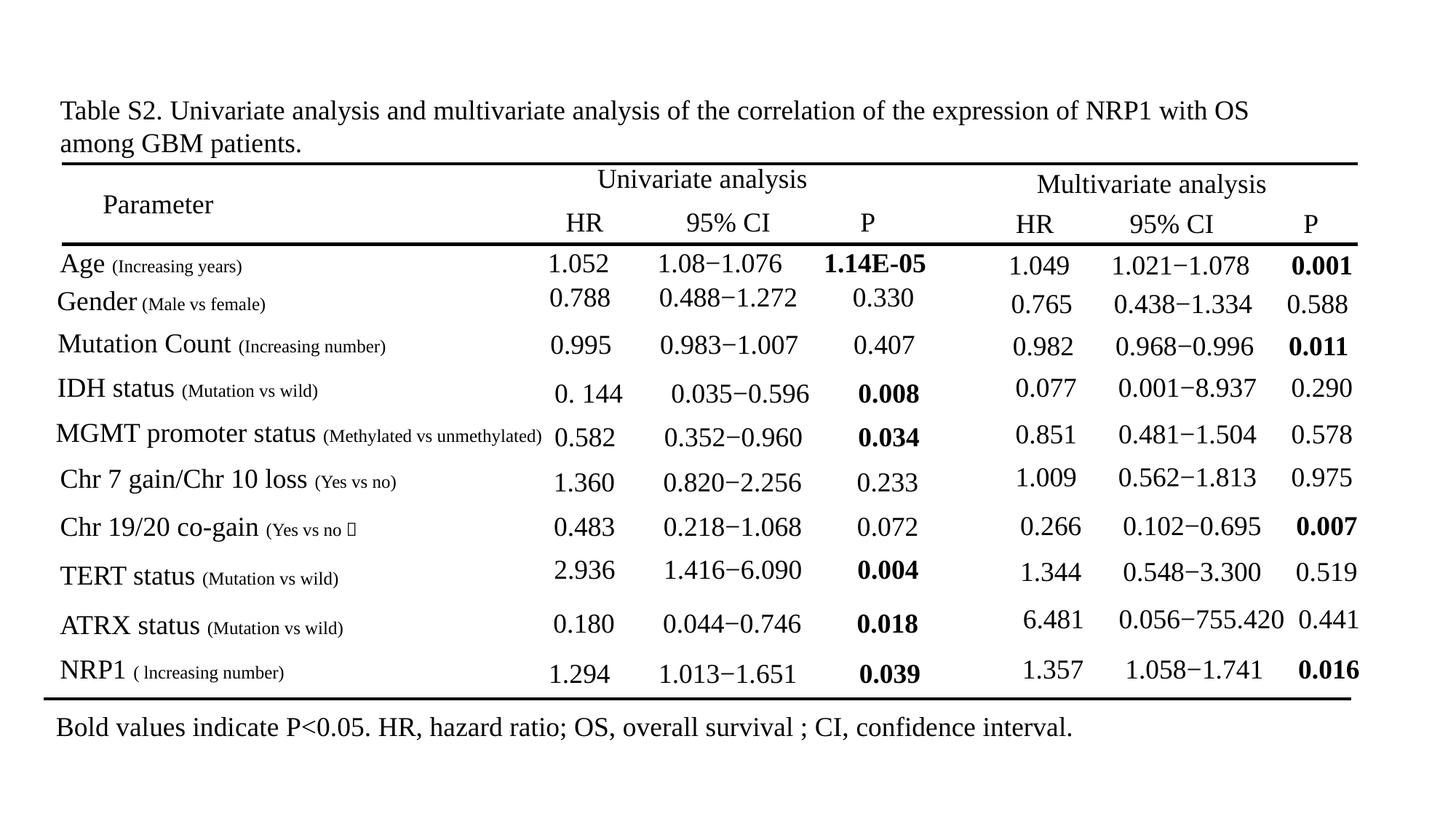

Table S2. Univariate analysis and multivariate analysis of the correlation of the expression of NRP1 with OS among GBM patients.
Univariate analysis
Multivariate analysis
Parameter
HR 95% CI P
HR 95% CI P
Age (Increasing years)
1.052 1.08−1.076 1.14E-05
1.049 1.021−1.078 0.001
0.788 0.488−1.272 0.330
Gender (Male vs female)
0.765 0.438−1.334 0.588
Mutation Count (Increasing number)
0.995 0.983−1.007 0.407
0.982 0.968−0.996 0.011
IDH status (Mutation vs wild)
0.077 0.001−8.937 0.290
0. 144 0.035−0.596 0.008
MGMT promoter status (Methylated vs unmethylated)
0.851 0.481−1.504 0.578
0.582 0.352−0.960 0.034
1.009 0.562−1.813 0.975
Chr 7 gain/Chr 10 loss (Yes vs no)
1.360 0.820−2.256 0.233
0.266 0.102−0.695 0.007
Chr 19/20 co-gain (Yes vs no）
0.483 0.218−1.068 0.072
2.936 1.416−6.090 0.004
1.344 0.548−3.300 0.519
6.481 0.056−755.420 0.441
0.180 0.044−0.746 0.018
ATRX status (Mutation vs wild)
NRP1 ( lncreasing number)
Bold values indicate P<0.05. HR, hazard ratio; OS, overall survival ; CI, confidence interval.
TERT status (Mutation vs wild)
1.357 1.058−1.741 0.016
1.294 1.013−1.651 0.039
